# Measuring working memory in contexts of high adversity: Using the digit span in North Kivu

**DOI:** 10.4102/ajopa.v7i0.177

**Published:** 2025-09-15

**Authors:** Isabelle Blanchette, Blaise Balume Bakulikira, Marie-Chantal Ingabire, Eric Kankunda Moket, Serge Caparos

**Affiliations:** 1 School of Psychology, Faculty of Social Sciences, Laval University, Québec, Canada; 2 Cervo Brain Research Center, Québec, Canada; 3 Department of Psychology, University of Goma, Goma, Democratic Republic of the Congo; 4 Department of Psychology, DysCo Laboratory, University Paris 8, France

**Keywords:** executive function, short-term memory, democratic Republic of Congo, cognition, armed conflicts, natural disasters

## Abstract

Having good measures of executive functions in general and of working memory (WM) in particular is very important, as these cognitive functions are associated with numerous consequential outcomes. Because few studies have examined WM in underprivileged populations exposed to high levels of adversity, including chronic armed conflicts and frequent natural disasters, the relevance of WM assessments in this context is still unknown. Our general objective was to examine the usefulness and usability of simple digit span measures of WM in contexts of high adversity, in North Kivu, in the eastern Democratic Republic of Congo, specifically by investigating links with formal schooling and other cognitive measures, as well as test–retest reliability. We conducted two studies. The first study included 97 internally displaced participants recently exposed to an upsurge in armed conflicts. In the second study, 281 participants were tested shortly after the eruption of the Nyaragongo volcano, and 115 of them were tested again 8 weeks after. We used the forward digit span, backward digit span and instruments measuring functional impairments and everyday cognitive skills. Performance on the digit span was associated with the level of schooling as well as everyday mathematical problem-solving and self-reported functional cognitive impairments. The results also showed a relatively strong stability of the forward digit span scores over an 8-week period.

**Contribution:**

Results of these studies support the usefulness of a simple digit span measure to study one important aspect of executive function in contexts of high adversity.

## Introduction

Executive functions (EFs) are a set of core cognitive functions that allow the integration and use of information for planning and self-directed behaviour (Cristofori et al., 2019). Until recently, EFs had mostly been studied in high-income countries, in highly schooled populations living in safe contexts. The knowledge derived from these samples and contexts cannot necessarily be assumed to apply universally. For cognitive research to be more inclusive, central constructs such as EF need to be empirically investigated in diverse populations. A growing number of studies demonstrate the importance of EF in a wider range of settings, including low- and middle-income countries in Africa (see, e.g. Bemath et al., 2020; Cockcroft, 2022). A cardinal EF is working memory (WM): the ability to store, manipulate and use information mentally over a short period (Postle & Pasternak, 2009). Executive functions also include cognitive flexibility and inhibition (Diamond, 1998). In this paper, we add to the growing literature on adult EF in Africa by exploring the usefulness of one short and easy-to-use measure of WM, the digit span, in contexts of high adversity.

Executive functions allow complex cognitive processes and are related to important life outcomes. Studies in Africa document how EFs can be affected by physical diseases, such as human immunodeficiency virus (HIV) infection (Akolo et al., 2014; Kanmogne et al., 2018), neurological disorders and how they tend to deteriorate with age (Kirova et al., 2015; Rabinovici et al., 2015). They are also related to mental health, psychiatric disorders and trauma exposure (Malarbi et al., 2017; Scott et al., 2015). Executive functions predict resilience after trauma (Bemath et al., 2020). Thus, EFs are both impacted by adversity and can shape how people adapt when facing difficult circumstances.

Most of the research on EFs generally, and on WM specifically, comes from minority-world countries (Kusi-Mensah et al., 2022; Schirmbeck et al., 2020). Nevertheless, a growing amount of work supports both the theoretical and empirical value of the concept of WM in African settings. For instance, the key conceptual distinction between verbal and visuo-spatial WM, proposed across a number of theoretical models of WM and empirically supported in numerous studies in the West (Postle & Pasternak, 2009), has been confirmed in African studies with undergraduate university students (Cockcroft, 2022; Wray et al., 2020). There is also evidence to support the important distinction between information storage and manipulation.

One key empirical feature of EF in general, and WM in particular, is that it should be related to schooling. Greater executive functioning, measured with batteries of tasks that include WM and other measures, favours success in school (Masten et al., 2012), and school attendance can improve at least some EF (Brod et al., 2017; Fuhs et al., 2018). Working memory, specifically measured with the digit span, similarly correlates with schooling, beyond chronological age (Roberts et al., 2015). Existing African studies have documented a link between EF and schooling in children or young adults (Bemath et al., 2020; Cockcroft, 2022; Finch et al., 2022; Wray et al., 2020). The few studies that exist with adults have focused on highly educated young adults (university students). Little is known about WM functioning in adults with lower levels of schooling in Africa, a fact that may be particularly important in a continent where access to and quality of education are highly variable (UNESCO Office Dakar and Regional Bureau for Education in Africa & Union Africaine, 2023).

Working memory measures are expected to be intercorrelated and to be related to other EF measures as well as to other complex cognitive functions such as reasoning and problem-solving (Anderson, 2002). There is some evidence of this in African studies (Bemath et al., 2020; Cockcroft, 2022; Wray et al., 2020). Other studies with African samples show positive correlations between WM and complex cognitive tasks, including reasoning and planning (Ainamani et al., 2017; Blanchette et al., 2019). Few studies have examined WM in populations facing extreme levels of adversity (Blanchette et al., 2019). Examining the link between WM task performance and other complex cognitive processes in a sample facing high levels of adversity is one of the goals of this study.

Because of its centrality as an important EF, WM should be related to everyday cognitive functioning that requires complex cognition, including planning, problem-solving or practically relevant mathematical operations. While there is some evidence of links between WM task performance and more ecologically valid measures of cognition in diverse samples (see for instance Depp et al., 2012; Ponsoni et al., 2023), to our knowledge, this has not yet been examined in Africa.

The digit span is a good candidate measure of WM to be used in diverse settings for several reasons. Measures of cognitive function that include semantic content (words, images, etc.) are difficult to use across diverse groups because of differences in language, culture and semantic knowledge (Evans, 2022). Digits are universally known, even in populations with very low levels of schooling (though the number of different digits may need to be adjusted – see Jukes & Grigorenko, 2010). Digits can be universally translated (though the length of pronunciation of each number in different languages needs to be considered, Chen & Stevenson, 1988). Another advantage of the digit span is that it is relatively simple and quick to administer. Research assistants can become competent at administering the task with reasonably little training. The digit span does have limitations. Its ecological validity appears limited, at face value; participants may not see the relevance of remembering arbitrary sequences of digits for their everyday lives. Nevertheless, if the task adequately assesses WM capacity, it may have very good predictive value for more ecological contexts and other complex cognitive tasks. One key aspect is that, in addition to correlating with other measures of cognition, a valid measure of WM must show good test–retest value over time (Waters & Caplan, 2003).

The general goal of our studies was to explore the validity and usefulness of a measure of verbal WM in an underprivileged population facing high levels of adversity. We use the digit span, a task in which participants must repeat sequences of digits in the same or in reverse order. Difficulty levels increase with added digits. The backward digit span involves both the storage and manipulation components of WM. The forward span focuses more exclusively on storage and is considered by some a component of WM and by others a separate construct of short-term memory or attention (LaBelle et al., 2019). Empirically, performance on the two tasks is correlated and represents both shared and independent variance (Gignac et al., 2018; Heled, 2024).

Our specific objectives were to (1) examine the link between performance on the digit span and schooling, (2) examine the link between performance on the digit span and measures of everyday cognitive functioning (context-relevant mathematical cognition and self-reported measures of cognitive difficulties) and also (3) examine the test–retest correlation of digit span performance (Study 2).

We hypothesised that (1) participants who attained higher levels of schooling would perform better on the backward and forward digit spans. We also hypothesised that (2) performance on the digit span would be negatively correlated with everyday cognitive difficulties and positively correlated with everyday mathematical cognition. We also hypothesised that (3) there would be a positive correlation between scores on a digit span task conducted several weeks apart. These outcomes would support the usefulness of the digit span task as a measure of WM, one aspect of EF.

### Context and overview of the studies

We conducted two studies in the eastern Democratic Republic of Congo (DRC). This region has been plagued by numerous natural disasters and chronically exposed to violence and armed conflicts since 1994 (Autesserre, 2010). It is also one of the poorest regions globally, as measured by the global Multidimensional Poverty Index, which considers health, education and living standards (Oxford Poverty and Human Development Initiative, 2021). In Study 1, we tested individuals who had recently been internally displaced following an upsurge in violence linked to the activity of the M23, an armed militia group formed in 2012. In June 2022, the group attacked villages in the Rutshuru region, resulting in 34 casualties, injuring 100 more people and forcing 165 427 people to flee (Fessy, 2022). We tested WM using a backward digit span, numeracy, mathematical problem-solving and daily problems related to cognitive difficulties and asked participants to report the highest level of formal schooling completed (primary, secondary, university).

We conducted Study 2 in Goma shortly after the eruption of the Nyiragongo volcano in May 2021. This event caused extensive damage (3500 houses, 7 schools and 4 health centres were destroyed and 200 000 people lost access to drinking water) and forced the displacement of 234 000 people (UNICEF, 2021). Latent underground gases in Lake Kivu resulting from the eruption threatened millions of people and maintained a high threat level for several weeks after the eruption. In Study 2, we measured verbal storage, one component of WM, using the forward digit span task, and asked participants to report the level of formal schooling achieved. We were able to test a subset of participants a second time, after a period of 8 weeks, to examine the test–retest reliability of the forward digit span in this second study.

## Study 1

### Methods

#### Context, recruitment and participants

This study was carried out in the territory of Rutushuru, in the province of North Kivu in the eastern DRC, between 12 July 2022 and 30 July 2022. It was part of a larger project aiming to examine the impact of recent exposure to armed conflicts on mental health and cognitive function (Blanchette et al., in press). In the present article, we report the results related to the digit span measure. The other article focuses on results relating to mental health and current living conditions.

Trained research assistants (4 women and 2 men) were supervised by two experienced local researchers (from the University of Goma, 2nd and 4th authors on this article). Researchers obtained permission from the local authorities to approach adults in two communities. Information about the study was disseminated through word of mouth. The researchers explained the general aspects of the task orally to interested individuals in small groups. Individual testing sessions were held in a private room or remote outside locations where participants would not be overheard or distracted. Research assistants read the information letter out loud to participants, answered any questions and obtained signed consent. Participants were compensated the equivalent of $2.50 for their time, which is approximately equivalent to the average daily salary in the DRC (World Bank, 2024). Data collection was conducted in Swahili. Completion of the study took approximately 40 min. We used the Kobo Toolbox instrument to programme the tasks and questionnaires and collect the data (see https://www.kobotoolbox.org/). The task can be shared with interested readers, who should contact the first author.

Our sample included 125 adult participants (18 years and older). The majority, 97 (76%), were internally displaced persons (IDPs) and 27 (24%) were from the host community (missing data on this question for 1 person). The participant characteristics are presented in Table 1. There was a majority of women, and the median age was 26.0 years. It must be noted that at times, age was approximated, as not all individuals knew their exact year or date of birth.

**TABLE 1:** Sample characteristics.

Variable	Study 1 (*n* = 125)				Study 2 – Time 1 (*n* = 281)				Study 2 – Time 2 (*n* = 115) subsample			
	Mean	s.d.	*n*	%	Mean	s.d.	*n*	%	Mean	s.d.	*n*	%
Age (years)	29.82	12.83	-	-	36.22	13.22	-	-	36.30	12.72	-	-
Lifetime trauma exposure	6.58	5.00	-	-	11.22	5.51	-	-	9.00	5.09	-	-
**Schooling level completed**												
None	-		82	66	-	-	48	30	-	-	24	34
Primary	-	-	39	31	-	-	80	50	-	-	36	51
Secondary	-	-	9	7	-	-	24	15	-	-	9	13
University	-	-	0	0	-	-	8	5	-	-	1	2
**Gender**												
Women	-	-	89	71	-	-	246	87.5	-	-	101	87.8
Men	-	-	34	27	-	-	35	12.5	-	-	14	12.2

s.d., standard deviation.

Participants were asked in three separate yes or no questions whether they had completed primary school, secondary school and university level. Only a minority of participants (see Table 1) had completed primary school. Very few participants reported having completed secondary education (7%) and none had a university degree.

Almost half of the sample (*n* = 57, 46%) reported that they had no income. Approximately, a quarter (*n* = 27, 22%) reported a monthly income below $20.00, and a similar proportion reported a monthly income between $20.00 and $100.00 (*n* = 20).

Most of our sample had been exposed to many potentially traumatic events both before and after the M23 attacks, confirming that this is a group of individuals facing high levels of adversity. Since the onset of the attacks (between 3 weeks and 6 weeks), participants had been exposed to about 8 (mean [*M*] = 7.9, standard deviation [s.d.] = 4.9) of the 25 potentially traumatic events that we selected from Part I of the Harvard Trauma Questionnaire (Mollica et al., 1992). Events include witnessing armed combat, having to flee and being tortured. The level of recent trauma exposure in our sample was high and higher in IDPs (*M* = 9.1, s.d. = 4.5) than in hosts (*M* = 3.6, s.d. = 4.1), *t*(42) = 5.96, *p* < 0.01, 95% confidence interval [CI]: 3.64 – 7.37, *d* = 1.29. More than half of the displaced individuals reported experiencing the following events since the attacks: Having their belongings confiscated, poor health with no access to medical care, having to hide or flee, having their house pillaged, witnessing armed combat, witnessing torture or witnessing the violent death of a loved one. Full details of trauma exposure and its connection to Post-Traumatic Stress Disorder (PTSD) and functional measures are reported in another article (Blanchette et al., in preparation).

### Instruments

All tasks were conducted in Swahili. Original instructions or contents were translated by expert local researchers and back-translated into French for verification.

**Backward digit span task:** We created a digit span task that was administered orally. Experimenters asked participants to remember sequences of digits and repeat them in reverse order. The experimenter explained the task and gave two examples for practice, insisting that for the answer to be correct, the right numbers needed to be provided in the right order. Experimenters also indicated that the difficulty level would increase progressively. The task started with sequences of 2 digits, increasing up to 8, each with two trials. If there was at least one correct answer, the next level was presented. With two incorrect trials, the task stopped.

Working memory performance was indexed in two ways. We report the backward span, which is the highest level for which a participant got at least 50% (one) sequence correct; this could span from 0 (minimum [min]) to 8 (maximum [max]). We also used the backward score, which simply grants one point for each correct response, multiplied by the level of difficulty. This score could span from 0 (min) to 16 (max).

#### Numeracy

We used an adaptation of the Test Diagnostic des compétences en mathématiques (TEDI-Math) (Grégoire et al., 2001) to measure numeracy. We asked participants to determine which of two numbers, presented orally, was the biggest. The instruction included an example (*If I tell you 1 and 2, you will tell me 2 is the bigger number*). Six pairs of numbers were presented, increasing in difficulty. The dependent measure was the sum of correct answers.

#### Mathematical problem-solving

A problem-solving task (also adapted from TEDI-Math [Grégoire et al., 2001]) was used to assess people’s ability to use mathematical concepts and numbers in everyday situations. The experimenter read aloud short problems such as this one: *Jean has 8 stones. He buys 6 more. How many stones does he have in total?* Five problems were presented, increasing in difficulty. The items were selected based on local expert knowledge and pilot tests; content was adapted culturally (for instance, by referring to pumpkins rather than peaches).

#### Daily Living Questionnaire

We adapted the Daily Living Questionnaire (DLQ) – Part I (Rosenblum et al., 2017), which we used as a measure of functional cognitive impairment. We selected 10 of the most relevant items (see Table 2) from the original 52 and translated them into Swahili. The items were also back-translated, and any discrepancy with the original items was resolved. This short version presented good internal consistency (Cronbach’s alpha = 0.80).

**TABLE 2:** List of items selected from the Daily Living Questionnaire to measure cognitive functional impairment.

Number	Backtranslation in English	Presented item in Swahili
1.	Clean the house or do the laundry	*Kuandaa kazi za nyumbani (kuandaa nguo)*
2.	Manage and spend money well	*Kuandaa na kutumiya vizuri pesa (franga)*
3.	Be able to find things in the cupboard	*Kupata vitu kwenye kwenye fasi inawekewa au chumbani*
4.	Participate in social activities with other people	*Kushiriki katika shughuli za kijamii na watu wengine*
5.	Participate in group discussion	*Kushiriki katika mijadala ya kikundi*
6.	Express your thoughts	*Kueleza mawazo yako*
7.	Plan and prepare meals	*Kupanga na kuandaa chakula*
8.	Remember where your things are	*Kukumbuka nafasi ambapo vitu vyako viko*
9.	Understand new information	*Kuelewa habari mpya*
10.	Remember the tasks you must do during the day	*Kukumuka nafasi ambapo vitu vyako viko wapi*

Participants were told that every day, we perform activities that require mental effort (or cognition) and that at some point people may experience mental or cognitive difficulties that make it more difficult to perform these tasks. Participants indicated the extent to which they experience mental or cognitive difficulty while performing each task. Higher scores indicate more difficulty (no = 1, some = 2, much = 3, unable to perform the task = 4).

#### Harvard Trauma Questionnaire

We used the event checklist from the Harvard Trauma Questionnaire (Mollica et al., 1992) to obtain a portrait of the level of adversity experienced by participants in our sample. The Harvard Trauma Questionnaire is a standardised instrument designed to assess trauma exposure, particularly in refugee populations. It presents a list of 25 potentially traumatic events and an open-ended question about the most distressing experience. The questionnaire has been adapted for various cultural contexts, ensuring its relevance across diverse populations. We used the existing validated Swahili version. Scores represent the sum of different events experienced by each participant.

### Statistical analyses

To examine the link between schooling and WM performance (Aim 1), we used independent samples *t*-test to compare the performance of participants who had completed primary education and those who had not. To determine the link between performance on the different cognitive tasks and self-reported cognitive difficulties in daily life (Aim 2), we used Pearson correlations. The significance level used was 0.05.

### Results

**FIGURE 1:**
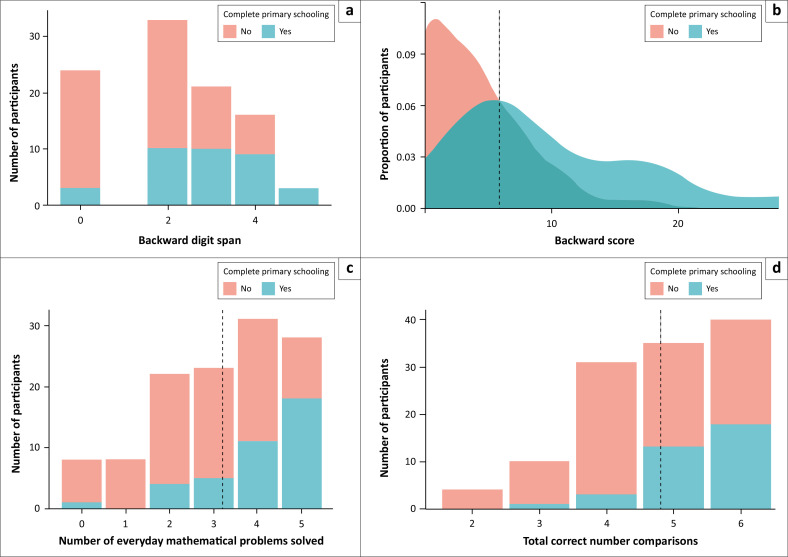
Main cognitive outcome variables in Study 1 as a function of whether participants completed primary school or not. Participants completed a backward digit span task starting with 2-digit sequences, increasing in length with correct answers. The backward span (a) represents the longest sequence with at least one out of two correct answers. The backward span score corresponds to the number of correctly recalled sequences multiplied by their length (b). The figure also presents the total number of correct answers on the mathematical problem-solving tasks (c) out of five items and on the number comparison task (d) out of six items. The mean for each variable is indicated by the grey vertical lines.

Figure 1 provides the distribution of scores and central tendency for four variables of interest as a function of schooling: backward span, backward score, mathematical problem-solving and number comparison. The range of scores shows that performance levels are broadly distributed.

There is a clear effect of schooling for all variables. Complete descriptive statistics of cognitive performances are presented in Table 3 separately for each level of schooling. Participants who had completed primary school had better scores than those who had not on the backward span (*t*(95) = 15.26, *p* < 0.001, *d* = 0.86), backward score (*t*(95) = 24.95, *p* < 0.001, *d* = 0.97), problem-solving (*t*(118) = 20.22, *p* < 0.001, *d* = 0.90) and number comparison (*t*(118) = 22.41, *p* < 0.001, *d* = 0.98).

**TABLE 3:** Descriptives of the performance on each of the cognitive measures, Study 1.

Measure	Schooling	*n*	Mean	s.d.	Min	Max
Backward digit span	Primary not completed	62	1.73	1.39	0	4
	Primary completed	35	2.89	1.30	0	5
Backward digit span score	Primary not completed	62	3.79	4.05	0	18
	Primary completed	35	9.54	7.31	0	28
Mathematical problem-solving	Primary not completed	81	2.81	1.46	0	5
	Primary completed	39	4.03	1.20	0	5
Number comparison	Primary not completed	81	4.51	1.11	2	6
	Primary completed	39	5.44	0.75	3	6
Daily living questionnaire	Primary not completed	82	18.16	8.46	0	40
	Primary completed	39	16.03	7.11	0	25

s.d., standard deviation; Min, minimum; Max, maximum.

**FIGURE 2:**
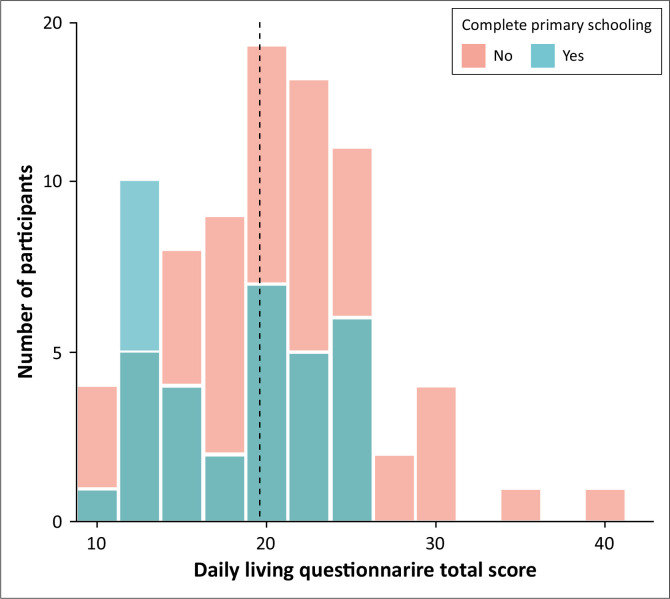
Total scores on the measure of cognitive functional impairments, adapted from the Daily Living Questionnaires. Scores can range from 10 (no difficulty performing any of the tasks presented in the 10 items) to 80 (unable to perform any of the tasks). Scores are presented separately for participants with completed primary schooling or not. The average is indicated by the grey vertical line.

Figure 2 shows that the observed scores on the Daily Living Questionnaire (DLQ) spanned the entire possible range (from 10 to 40), with four people reporting never having any difficulty and one person reporting not being able to do any of these tasks. The scores were relatively evenly distributed, but with fewer participants reporting difficulty on more than half of the items. DLQ scores did not significantly differ between participants who had completed primary school and those who had not (*t*(119) = 1.8, *p* = 0.17, *d* = 0.27).

**FIGURE 3:**
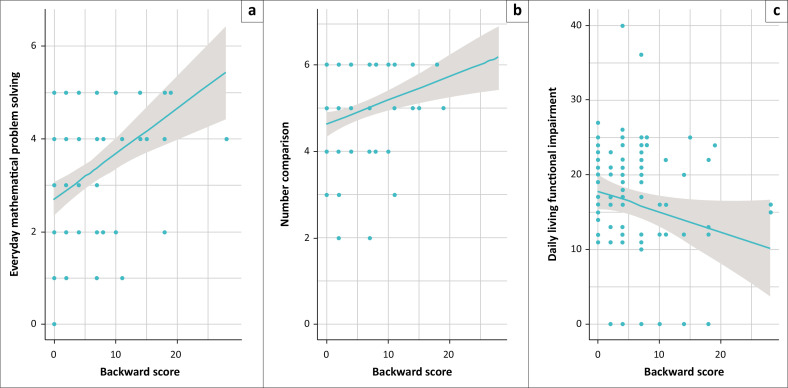
Correlations between the backward score, which corresponds to the number of correctly recalled sequences in the backward digit span, multiplied by their length, and total number of items solved in the mathematical problem-solving task (a), the number comparison task (b), and total scores on our adaptation of the Daily Living Questionnaire (possible range = 10–80), which measures cognitive functional impairment, where higher scores represent more difficulty. Individual observations are presented as data points, along with regression lines and the grey area represents the 95% confidence interval.

Finally, we examined the correlations between backward scores and other cognitive tasks (see Figure 3). Backward scores were positively and significantly related to mathematical problem-solving (*r* = 0.40, 95% CI = 0.23; –0.56, *p* < 0.001) and number comparison (*r* = 0.32, 95% CI = 0.14; –0.49, *p* < 0.001). Participants who had better WM performed better on these two measures of contextualised cognition. The negative link between backward scores and the DLQ was marginally significant (*r* = –0.19, 95% CI = –0.37; –0.06, *p* = 0.057), suggesting that this WM measure may also be related to self-reported cognitive difficulties in everyday life.

### Discussion

In Study 1, we explored the usability and validity of a short measure of verbal WM with participants who experience high levels of adversity. Difficulty levels on the different tasks seemed appropriate for this sample with low levels of schooling. Results suggest that our instruments tapped into important dimensions of individual differences in cognitive functioning.

Results of Study 1 support the usability and validity of the backward digit span as a measure of WM. There was a strong link between WM and formal schooling as well as with all performance-based measures. There were also small to moderate correlations between WM and indicators of everyday cognitive functioning – both performance-based (number comparison and problem-solving) and self-reported, though the latter was not statistically significant.

In Study 2, we use the forward digit span, a measure more specifically linked to verbal storage (Baddeley, 2010). Our goal was to replicate the correlation between WM and schooling in a different sample and different context of adversity and to examine the stability of the measure over time.

## Study 2

### Methods

#### Context, recruitment and participants

This study was carried out in the Nyiragongo region and Goma, shortly after the eruption, on 22 May 2021, of the Nyiragongo volcano. The study was conducted entirely in Swahili.

Data for the Time 1 test were collected between 21 July 2021 and 10 August 2021, within 50 days of the volcanic eruption. At this time, IDPs were living in camps and with host families. Humanitarian aid was negligible. Time 2 testing was conducted on average 8 weeks later. Conditions had improved, and some people had returned to their homes, but some were still living in camps in difficult conditions with little assistance.

Recruitment proceeded as in Study 1, involved the same local researchers and the research assistants were trained in the same way. Participants took approximately 45 min to complete the study.

Our sample included 281 participants at Time 1, and 115 participants at Time 2. Sample characteristics for the complete sample (Time 1) and the subset of participants who also completed Time 2 are presented in Table 1. It can be seen that the subsample is very similar to the general sample. There was a majority of women at both times, and the median age was 36 years old at both times.

One hundred and eighty-three participants were directly exposed to the eruption and displaced. The remaining 92 participants were more distant from the eruption (1 km – 2 km). Participants directly exposed did not report a significantly greater number of lifetime stressors (*M* = 11.50, s.d. = 5.73) compared to those indirectly exposed (*M* = 10.80, s.d. = 6.08), *t*(203) = 0.99, *p* = 0.31, *d* = 0.18, as measured by the Harvard Trauma Questionnaire (Mollica et al., 1992). When asked to report stressors since the eruption, most of the participants directly exposed reported being homeless, lacking food or water, experiencing poor health without access to health care, being forced to flee and having their property destroyed since the eruption of the volcano.

Participants were asked their level of schooling with an open-ended question (*What is the highest level of schooling you have achieved*?) and responses were coded by local experts to map onto the categories used in Study 1. The answer to this question was missing for a lot of participants (*n* = 121). The distribution of answers is presented in Table 1. Because there were very few participants who attended university, these participants were combined with those who completed the secondary level for statistical analyses (forming a ‘Secondary +’ category).

### Instruments

#### Forward digit span

To measure verbal storage, which is one component of WM, we asked participants to remember sequences of digits and repeat them in the same order. In all other respects, the procedure was the same as in Study 1. Sequences went from 2 to potentially 8 digits. Again, we used two dependent measures: forward span (the longest sequence with at least one correct answer) and forward score (the sum of accurate answers multiplied by the length of the sequence).

### Statistical analyses

We used the same strategy as in Study 1 to examine the link between schooling and WM performance (Aim 1): we used independent samples *t*-test to compare the performance of participants who had completed primary education and those who had not. To determine test–retest validity (Aim 3), we used Pearson correlations. The significance level used was 0.05.

### Results

**FIGURE 4:**
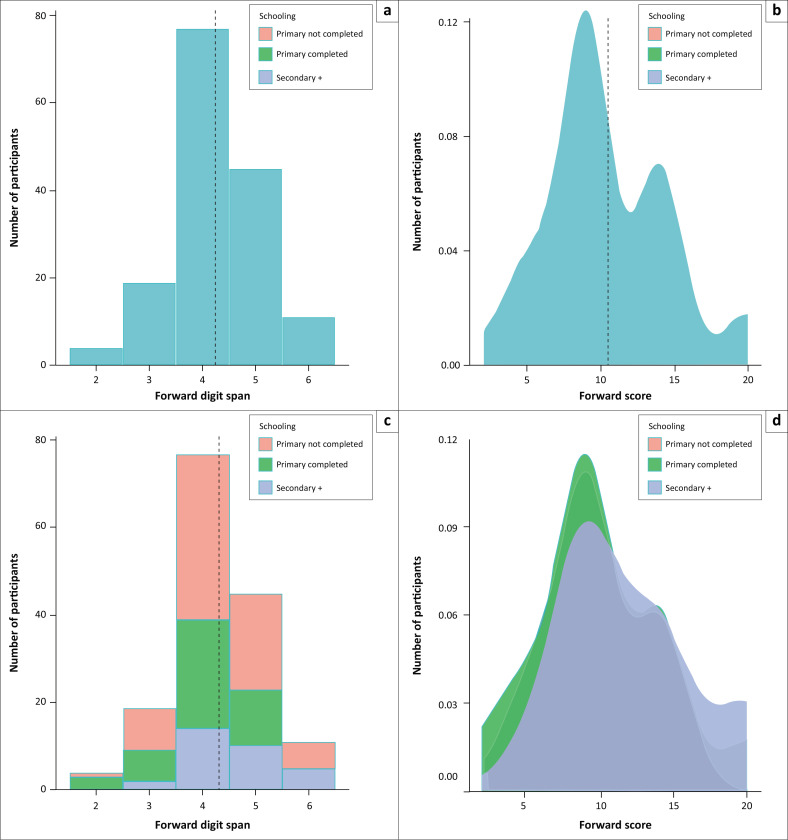
Presentation of forward digit span outcome measures in Study 2 for the entire sample (a and b, *n* = 281) and for participants for whom we had information about schooling level (c and d, *n* = 160). The forward digit span task started with 2-digit sequences, increasing in length with correct answers. The forward digit span (a and c) represents the longest sequence with at least one out of two correct answers. The forward span score (b and d) corresponds to the number of correctly recalled sequences multiplied by their length. The bottom panels present the data separately for participants with different levels of schooling. Dotted lines represent the overall average.

Figure 4 presents the distribution of the scores, showing the range, variability and central tendency (mean represented by the grey vertical line).

The mean forward span (*F*(2, 140) = 3.61, *p* < 0.05, *d* = 0.67) and the forward score (*F*(2, 140) = 3.38, *p* < 0.05, *d* = 0.69) differed as a function of the level of schooling. Participants with completed primary (*M* = 10.50, s.d. = 4.14) and some secondary education (*M* = 12.00, s.d. = 4.43) had higher forward scores than those without a completed primary education (*M* = 9.33, s.d. = 3.51).

We examined the correlations across time for participants who had completed both measures (*n* = 135). Forward scores at Time 1 were positively and significantly correlated with scores at Time 2 (*r* = 0.43, 95% CI = 0.28–0.55, *p* < 0.001).

### General discussion

The general goal of this project was to examine the validity and usefulness of simple measures of verbal WM in underprivileged samples experiencing high levels of adversity. We found that the backward digit span (Study 1) and the forward digit span (Study 2) were effective measures of WM, addressing verbal information manipulation and simple storage, respectively. The two measures correlated with level of schooling (Aim 1). Performance showed significant inter-individual differences, and these correlated with other measures of cognition, including contextually relevant tasks (Aim 2). Digit span performance also showed stability over time, with relatively strong intra-individual correlations between two measures taken 8 weeks apart (Aim 3). Finally, the backward and forward and digit span tasks have undeniable practical advantages, including the possibility to modulate difficulty level, the ease of task presentation and the ability to easily translate instructions into several languages.

It is possible to criticise models of cognition for being Western-centric. Most of the empirical and theoretical work on EFs, including WM, comes from highly educated populations in rich countries. It is important to examine the validity of conceptual models and tasks as well as the replicability of results beyond minority-world settings.

One key theoretical and empirical feature is that WM should be correlated with schooling. Our first specific aim was to examine the link between digit span scores and schooling in a sample experiencing high levels of adversity, with overall relatively low levels of formal school completed. Across the two samples, we observed a link between performance on WM tasks and schooling level. These correlational data cannot unambiguously determine whether a better WM promotes school perseverance or whether school attendance develops WM. However, in the DRC, the level of schooling achieved is heavily determined by social and contextual challenges and resources. The large-scale insecurity that has plagued North Kivu in the last 25–30 years has had a determining impact on school attendance (UNICEF, 2023). Our results are consistent with the hypothesis that participants who achieved higher levels of school had a greater opportunity to develop their EFs. Longitudinal studies demonstrate a causal effect of school attendance on attentional control and other EFs (Kim et al., 2021). The effect of schooling that we observed seemed greater in Study 1 than in Study 2 (effect sizes 0.86–0.96 vs. 0.67–0.69). Individuals in the second study reported completing overall higher levels of school. The effects of schooling on MCT may be non-linear and may be more pronounced at the lower levels. Completing a primary education may have more impact on simple WM tasks such as the digit span than additional years in secondary education. The stronger link with schooling observed in Study 1 may also stem from the fact that this study used a backward digit span, which requires information manipulation, whereas Study 2 used a forward digit span, which is cognitively simpler and requires only information storage. Schooling may have more impact on more complex cognitive function. These questions should be explored in future studies. Future studies should also consider the quality of schooling, which could have a determining effect on cognitive development beyond simply the number of years in school. Nonetheless, our results demonstrate a strong link between schooling and WM, consistent with the results from studies in Western, Educated, Industrialised, Rich and Democratic (WEIRD) countries. This supports the validity of the digit span measures in high-adversity contexts.

Equally important, digit span performance was correlated with more ecologically valid measures of cognition. Our second specific goal was to examine the link between WM measures and other measures of complex cognitive function, including more ecologically valid tasks, something that had yet to be observed in this type of sample exposed to high levels of adversity and with relatively low levels of schooling. The number comparison and problem-solving tasks, as well as self-reported cognitive difficulties, were measured to provide an indication of the use of complex cognitive functions in everyday life. While the difficulty level of the behavioural tasks (number comparison and mathematical problem-solving) seemed adequate, the self-reported questionnaire could be improved to include more complex items. This may explain the low correlation between scores on the adapted Daily Living Questionnaire and digit span performances. Nevertheless, simply observing a link between a performance-based measure of WM and functional measures is very important and, to our knowledge, a novel finding in an African context.

Our study has several limitations. While the digit span task provides valuable information about WM, one dimension of EF, it does not capture the full complexity of EF. Future studies may include a more comprehensive evaluation of EF, using a battery of measures to also assess inhibition and cognitive flexibility. We measured information manipulation and storage, consistent with a recognised definition of WM (Postle & Pasternak, 2009). However, there is a debate about whether simple verbal storage should be considered part of WM. We used different measures of schooling across the two studies. Finally, most of the effects tap a relatively small portion of the variance.

Despite these limitations, our results demonstrate the relevance of using a simple measure of WM to study EF in contexts of high adversity, with populations presenting low or highly variable levels of schooling. Measuring cognition provides crucial information to understand coping following adversity and in challenging contexts.
